# Silva cumulative score and its relationship with prognosis in Endocervical adenocarcinoma

**DOI:** 10.1186/s12885-022-10270-7

**Published:** 2022-11-14

**Authors:** Yuemin Li, Meng Jia, Lanqing Cao, Jiaqi Yu, Hongwen Gao, Ping-Li Sun

**Affiliations:** Department of Pathology, The Second Hospital of Jilin University, 218 Ziqiang Road, Changchun, 130041 Jilin China

**Keywords:** Endocervical adenocarcinoma, Pattern-based classification, Prognosis, Silva classification system

## Abstract

**Background:**

The Silva system has been demonstrated to have a good predictive value of lymph node metastasis (LNM) in endocervical adenocarcinoma (EAC). Tumours were classified based on the highest identified pattern of invasion in this system, this may not exactly reflect the true situation when it presents with a “mixed pattern” in some cases. Recent study has shown that patients with lymphovascular invasion (LVI) have worse prognosis in EAC. Here we design a Silva cumulative score (SCS) system which also combined the LVI status to explore its prognostic role in EAC patients.

**Methods:**

A total of 120 patients with EAC were included in this study. Clinicopathological characteristics were retrospectively retrieved from the medical records and follow-up data were obtained. The clinicopathological information included age at diagnosis, depth of invasion (DOI), LNM, LVI, Silva classification, and SCS. SCS is a classification system based on the sum score of different Silva pattern which is founded on morphological phenomena. The relationships between the pathological characteristics and prognoses were analyzed.

**Results:**

According to the Silva system, 11 (9.2%), 22 (18.3%) and 87 (72.5%) patients had patterns A, B, and C, respectively. Patients with pattern C had the highest incidence of LVI and LNM (*p* < 0.05). Although the Kaplan-Meier curves demonstrated that survival decreased with increasing Silva classification for A-C cancers, there was no statistically significant difference [disease-free survival (DFS): *p* = 0.181; overall survival (OS): *p* = 0.205]. There were 45 cases presented as mixed-type of Silva patterns. According to the SCS, 23 cases (19.2%) were rated as grade I, 31 cases (25.8%) as grade II and 66 (55.0%) cases as grade III. Patients with SCS grade III had the highest incidence of LVI and LNM (*p* < 0.05). Kaplan–Meier analysis revealed that patients with higher SCS had significantly shorter DFS and OS than those with lower SCS (*p* < 0.05). High SCS was an independent predictor of poorer OS and DFS (*p* < 0.05) in patients with EAC.

**Conclusions:**

The application of the Silva system could effectively predict the LNM of patients and may be helpful in selecting an appropriate surgical procedure. The SCS system we designed showed a good predictive value for DFS and OS in EAC.

## Background

Endocervical adenocarcinoma (EAC) is the second most common cervical cancer among women after cervical squamous cell carcinoma with an increasing incidence [1–5]. Elvio G. Silva created a new classification system for human papillomavirus (HPV) associated (HPVA) EAC based on the pattern of invasion in 2013 [6–10]. The Silva system has been demonstrated a good value for surgeons to choose an optimal surgical approach. Previous studies reported the recurrence rate and mortality rate in different Silva patterns [3, 6, 8, 11–19]. However, on account of the restricted follow-up period or small sample size, most literatures have not carried survival analyses, results from the limited number of studies which perform an analysis for long term survival have led to different conclusions [14, 15, 18–20].

Tumours are known to be heterogenous [21–23] and previous studies have showed that agreement between patterns in biopsy and the overall tumour was only 37.5% [12]. The existence of a “mixed pattern” of invasion is recognized by the original literature [6].And as a result, the Silva system requires to confirm the entire tumour. A tumour should be classified based on the highest identified pattern of invasion [6, 11] according to the existing criteria of the Silva system. This may not exactly reflect the true situation when it presents with a “mixed pattern” in some cases.

In previous studies which further distinguish different subtypes of pattern C [16, 24], it was found that even within pattern C, patients with different morphotypes, including mixed patterns, have different prognoses. In other words, when there are two different morphologies, the prognosis of the patients is different from those who have only the pure form. These suggest the possibility that cases present mixed Silva patterns may have different prognoses with others.

Lymphatic vascular infiltration (LVI) has been demonstrated a prognostic indicator of patient outcome in most types of cancer [25–27]. In the follow-up study [13] by team of Silva, it has been shown that group stratification based on LVI helped predict outcome in patients with pattern C tumour. Also, some recent papers demonstrated that Silva pattern and LVI are strong prognosticators especially in tumours with early stage [28, 29].

In this study, we attempted to propose a Silva cumulative score (SCS) system which incorporate a factor of morphologic heterogeneity. Considering the prognostic role of LVI in EAC [13, 28, 29], we integrated LVI in SCS system. Our results indicated that SCS may be valuable to predict overall survival (OS) and disease-free survival (DFS). In addition, this study also verified the guiding value of Silva system for surgical scheme through the retrospective analysis of EAC specimens.

## Methods

Clinical and pathological data of patients who met the criteria in The Second Hospital of Jilin University from January 2009 to December 2017 were collected and retrospectively analyzed. Tumours were classified according to the 5th edition World Health Organization (WHO) classification of Female Genital tumours [30]. The patients were restaged according the 2019 International Federation of Gynecology and Obstetrics (FIGO) staging guidelines [2, 31]. The follow-up data were obtained and defined as the time from the date of surgery to January 2020, and was recorded in months. OS was defined as the interval from the date of surgery until death from disease or the last follow-up date, whereas DFS was defined as the interval from tumour resection to disease recurrence, death, or last follow-up date. The inclusion criteria were as follows: (1) The pathological diagnosis of the patients was invasive EAC; (2) The patients who underwent radical hysterectomy; and (3) Patients with complete follow-up data.

All slides were evaluated by experienced pathologists who reviewed the slides together and reached a consensus, and pathological features were recorded as the follows: (1) Silva classification, according to the pattern-based classification standard of HPVA EAC in the 5th Edition WHO classification of Female Genital tumours [30]; (2) SCS (Table 1): Based on the morphological criteria of the Silva classification, we assign a score to each Silva pattern (1 for pattern A, 2 for pattern B, and 3 for pattern C). The SCS in this study includes a primary score and a secondary score, which are the numerical values for the two most prevalent differentiation patterns. The primary score was the one representing the majority of the lesion, whereas the secondary score represented the second most prevalent Silva pattern. The primary and secondary score were added to obtain the total lesion SCS. Only the highest score was retained if the worst pattern exceeded 80%. In addition, if LVI was identified in the case, 1 extra point was added. SCS were assigned to tumours as combinations of grade I (score 2–3, low aggressive), grade II (score 4–5, highly aggressive), and grade III (score 6–7, very highly aggressive). (3) Depth of invasion (DOI): According to the ratio of this depth to the thickness of the normal cervical wall, DOI was divided into<1/2 and >1/2. (4) LVI. (5) Pelvic lymph node metastasis (LNM).

**Table 1:** Grading rubrics of SCS

Grade	Score	Computational methods	Morphology by light microscopy
grade 1	2	1 + 1 = 2	Only pattern A, LVI negative
	3	1 + 2 = 3, 2 + 1 = 3	Mixture of pattern A and pattern B, LVI negative
grade 2	4	①1 + 3 = 4, 3 + 1 = 4	①Mixture of pattern A and pattern C, LVI negative
		②2 + 2 = 4	②Only pattern B, LVI negative
		③1 + 2 + 1 = 4, 2 + 1 + 1 = 4	Mixture of pattern A and pattern B, LVI positive^a^
	5	①2 + 3 = 5,3 + 2 = 5	At least pattern B and pattern C emerge (with or without pattern A), LVI negative
		②2 + 2 + 1 = 5	Only pattern B, LVI positive^a^
		③1 + 3 + 1 = 5, 3 + 1 + 1 = 5	Mixture of pattern A and pattern C, LVI positive^a^
grade 3	6	①3 + 3 = 6	Only pattern C, LVI negative
		②2 + 3 + 1 = 6, 3 + 2 + 1 = 6	At least pattern B and pattern C emerge (with or without pattern A), LVI positive^a^
	7	3 + 3 + 1 = 7	Only pattern C, LVI positive^a^

Silva A = 1, Silva B = 2, Silva C = 3, Only the highest score was retained if the worst pattern exceeded 80%. ^a^If LVI was identified in the case, 1 extra point should be added

Statistical analysis was performed using SPSS (IBM SPSS 20.0, SPSS Inc). Continuous variables were compared using one-way analysis of variance (ANOVA). Kruskal–Wallis test were utilized to compare the ordered categorical variables, whereas Pearson’s chi-square test and Fisher’s exact test were used to compare the unordered categorical variables. The log-rank test and Kaplan–Meier curves were used for the survival analysis. Multivariate survival analyses were performed using Cox proportional hazards model (Cox regression). Statistical significance was set at *p* < 0.05.

## Results

This study included 120 patients who were followed up, and the mean age at diagnosis was 48 years (range 26–73 years). Overall, LVI and LNM occurred in 52 (43.3%) and 24 (20.0%) patients, respectively. According to the FIGO staging system, 65 patients (54.2%) were stage I, 31 (25.8%) were stage II, and 24 patients (20.0%) were stage III.

### Analysis of general characteristics of patients according to Silva system

According to the diagnostic criteria of the Silva system, 11 patients (9.2%) were diagnosed with pattern A, included 10 cases (90.9%) of FIGO stage I and 1 case (9.1%) of FIGO stage II. None of the patients had LVI or LNM. Pattern B was identified in 22 cases (18.3%), of which 12 cases (54.5%) were FIGO stage I, 8 cases (36.4%) were FIGO stage II, and 2 cases (9.1%) were FIGO stage III. LVI were observed in 6 cases (27.3%) and LNM in 2 cases (9.1%). The highest incidence was pattern C with 87 cases (72.5%), 43 of them (49.4%) were FIGO stage I, 22 (25.3%) were FIGO stage II, and 22 cases (25.3%) were FIGO stage III. LVI and LNM was found in 46 (52.9%) and 22 (25.3%) patients, respectively. The differences in DOI, LVI, FIGO stage and LNM among the three patterns were statistically significant (*p* < 0.05). Patients with pattern C tumours had the most advanced FIGO stages, the highest incidence of LVI and LNM, and the highest DOI. There was no correlation of Silva classification with age (*p* = 0.541) or incidence of HSIL (*p* = 0.830). These results are shown in the Table 2.

**Table 2:** Correlation between clinicopathological parameters of 120 EAC patients and Silva system/SCS

clinicopathological characteristics	total	Silva system			*p*	Silva cumulative score			*p*
		pattern A	pattern B	pattern C		grade I (score2–3)	grade II (score 4–5)	grade III (score 6–7)	
n	120 (100)	11 (9.2)	22 (18.3)	87 (72.5)		23 (19.2)	31 (25.8)	66 (55.0)	
age									
median	48 (26–73)	46 (35–65)	45 (28–73)	49 (26–69)	0.541	46 (29–65)	46 (26–73)	51 (32–69)	0.050
average	47.98 ± 9.10	47.27 ± 10.46	46.18 ± 10.71	48.53 ± 8.52		46.35 ± 9.93	45.35 ± 9.63	49.79 ± 8.23	
with HSIL									
No	103 (85.8)	10 (90.9)	18 (81.8)	75 (86.2)	0.830	19 (82.6)	26 (83.9)	58 (87.9)	0.777
Yes	17 (14.2)	1 (9.1)	4 (18.2)	12 (13.8)		4 (17.4)	5 (16.1)	8 (12.1)	
DOI									
<1/2	51 (42.5)	10 (90.9)	13 (59.1)	28 (32.2)	0.000	20 (87.0)	14 (45.2)	17 (25.8)	0.000
>1/2	69 (57.5)	1 (9.1)	9 (40.9)	59 (67.8)		3 (13.0)	17 (54.8)	49 (74.2)	
LVI									
Negative	68 (56.7)	11 (100.0)	16 (72.7)	41 (47.1)	0.000	23 (100)	23 (74.2)	22 (33.3)	0.000
Positive	52 (43.3)	0	6 (27.3)	46 (52.9)		0	8 (25.8)	44 (66.7)	
FIGO stage									
I	65 (54.2)	10 (90.9)	12 (54.5)	43 (49.4)	0.025	17 (73.9)	21 (67.7)	27 (40.9)	0.001
II	31 (25.8)	1 (9.1)	8 (36.4)	22 (25.3)		6 (26.1)	7 (22.6)	18 (27.3)	
III	24 (20.0)	0	2 (9.1)	22 (25.3)		0	3 (9.7)	21 (31.8)	
LNM									
Negative	96 (80.0)	11 (100.0)	20 (90.9)	65 (74.7)	0.046	23 (100)	28 (90.3)	45 (68.2)	0.001
Positive	24 (20.0)	0	2 (9.1)	22 (25.3)		0	3 (9.7)	21 (31.8)	

### Analysis of clinicopathological characteristics of patients according to SCS

**Fig. 1:**
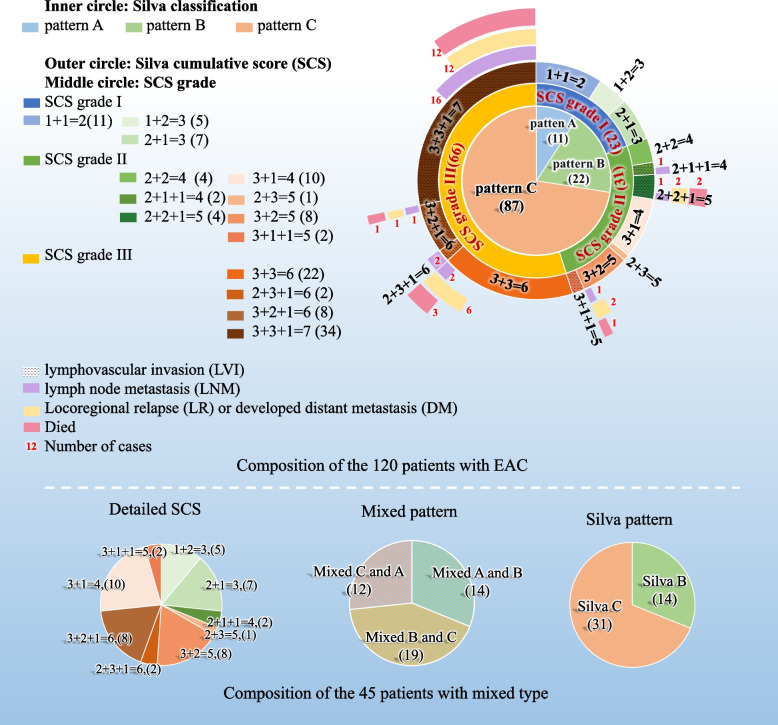
Contrast of Silva classification (inner circle), SCS grade (middle circle) and detailed SCS (outer circle). Blue lines represent pattern A in the Silva system; green lines represent pattern B; yellow lines represent pattern C. The numbers in parenthesis represent the number of cases in corresponding areas. For composition of the 45 patients with mixed type, pie charts are shown below the graph

Figure 1 summarizes the associations contrasting Silva classification and SCS. It showed that 37.5% (45/120) of the cases were mixed morphology. There were 14 (11.7%, 14/120) cases with mixed morphologies of Silva A and B, 19 (15.8%, 19/120) cases with mixed morphologies of Silva B and C, 12 (10.0%, 12/120) cases with mixed morphologies of Silva A and C. Using the criteria of SCS, 23 (19.2%, 23/120) cases of SCS grade I were identified which included all of the pattern A (100%, 11/11) and 12 cases (54.5%, 12/22) of pattern B in Silva system. Among them, 11 cases (9.2%, 11/120) were rated as score 2, and 12 cases (10.0%, 12/120) were score 3 (5 cases with 1 + 2, 7 cases with 2 + 1). No patient with SCS grade I had the presence of LNM. There were 31 patients (25.8%, 31/120) were rated as SCS grade II, of which 16 (13.3%, 16/120) were diagnosed as score 4 (4 cases with 2 + 2, 10 cases with 3 + 1, 2 cases with 2 + 1 + 1), and 15 (12.5%, 15/120) were score 5 (1 case with 2 + 3, 8 cases with 3 + 2, 4 cases with 2 + 2 + 1, 2 cases with 3 + 1 + 1). The 31 cases with SCS grade II contained a part of pattern B (45.5%, 10/22) and a part of pattern C (24.1%, 21/87). LNM occurred in three (9.7%, 3/31) cases. SCS grade III was diagnosed in 66 patients (55.0%, 66/120), all cases were pattern C, which represented 75.9% (66/87) of all cases of pattern C. 32 (26.7%, 32/120) of them were score 6 (22 cases with 3 + 3, 8 cases with 3 + 2 + 1, 2 cases with 2 + 3 + 1), 34 (28.3%, 34/120) of them were score 7 (3 + 3 + 1). 21 patients (31.8%, 21/66) had the presence of LNM. Similar to the Silva system, statistical analysis revealed remarkable differences between the three SCS grades in DOI, LVI, FIGO stage and LNM (*p* < 0.05). No remarkable differences were observed between SCS grade and age (*p* = 0.050) or incidence of HSIL (*p* = 0.777). Summary statistics and statistical analyses are in Table 2.

### Prognosis according to Silva system and SCS

All the 120 cases were followed up. The follow-up period ranged from 25 to 95 months, with a median follow-up of 54 months. Up to the last day of follow-up, 24 patients developed locoregional relapse (LR) or developed distant metastasis (DM), and 19 patients died. The 1-, 3-, and 5-year survival rates in the 120 cases were 91.7, 86.5 and 84.2%, respectively. According to the Silva system, the 1-, 3-, and 5- year survival rates were all 100% for pattern A, 100, 90.9, and 90.9% for pattern B, respectively, 96.6, 83.7, and 80.9% for pattern C. According to the SCS, the 1-, 3-, and 5- year survival rates were all 100% for SCS grade I, 97.4, 89.6 and 89.6% for SCS grade II, respectively, 94.6, 78.1 and 73.6% for SCS grade III.

### Analysis of prognostic factors of OS and DFS

**Fig. 2:**
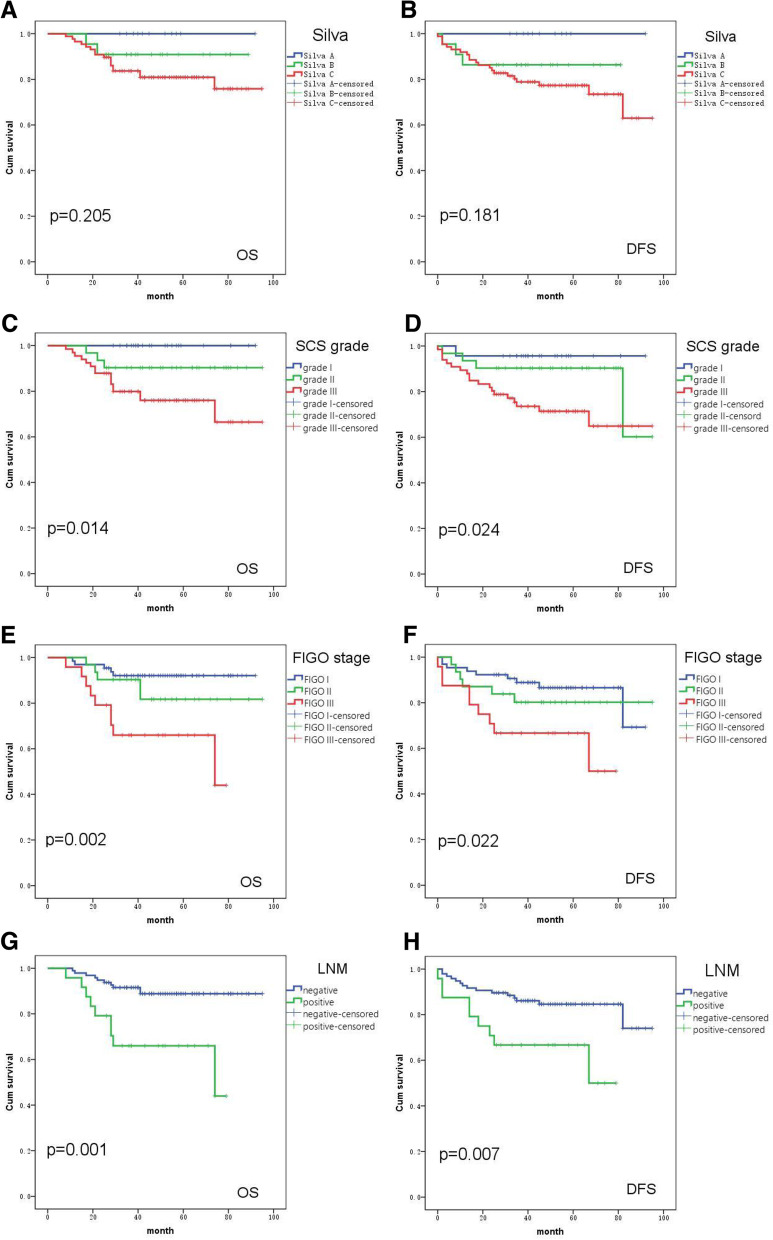
Kaplan-Meier curves of OS (left) and DFS (right) for 120 EAC patients. There was no significant difference in OS (**A**) and DFS (**B**) among the three groups based on the Silva system. Higher SCS grades were associated with shorter OS (**C**) and DFS (**D**). A higher FIGO stage was also associated with shorter OS (**E**) and DFS (**F**). There were significant differences in OS (**G**) and DFS (**H**) between the LNM-positive and LNM-negative groups

Univariate survival analysis (Table 3) showed that SCS grade, DOI, LVI, FIGO stage, and LNM were significantly associated with OS and DFS (*p* < 0.05), patients who had high SCS grade appeared to be significantly associated with worse DFS and OS than those who had low SCS grade. However, there was no association between OS and DFS with Silva system (OS: *p* = 0.205; DFS: *p* = 0.181). Kaplan-Meier survival curves are presented in Fig. 2. In multivariate analyses (Table 4), SCS was demonstrated to be an independent prognostic factor for OS (hazard ratio [HR] = 3.012, *p* =  0.049) and DFS (HR  =  2.626, *p* =  0.013) in HPVA EAC.

**Table 3:** Univariate analysis of the prognostic factors for survival

clinicopathological characteristics	OS (month)			DFS (month)		
	mean OS	95%CI	*p*-value	mean DFS	95%CI	*p*-value
FIGO stage						
I	86.404	81.673–91.134	0.002	80.687	73.876–87.498	0.022
II	83.097	73.588–92.605		79.356	68.054–90.659	
III	58.090	47.000–69.181		54.750	42.077–67.423	
Silva system						
pattern A	NA	NA	0.205	NA	NA	0.181
pattern B	NA	NA		NA	NA	
pattern C	NA	NA		NA	NA	
DOI						
<1/2	87.784	83.232–92.336	0.008	82.859	75.953–89.764	0.047
>1/2	77.488	69.949–85.026		72.234	63.135–81.333	
SCS grade						
grade I (score 2–3)	NA	NA	0.014	88.348	81.347–95.349	0.024
grade II (score 4–5)	NA	NA		82.860	72.364–93.357	
grade III (score 6–7)	NA	NA		71.109	61.975–80.244	
LVI						
Negative	90.817	86.852–94.782	0.000	84.004	77.212–90.797	0.021
Positive	72.566	63.005–82.126		69.811	59.064–80.558	
LNM						
Negative	87.262	82.714–91.809	0.001	81.841	75.678–88.004	0.007
Positive	58.090	47.000–69.181		54.750	42.077–67.423

**Table 4:** Multivariate analysis of the prognostic factors for survival

clinicopathological characteristics	OS (month)			DFS (month)		
	HR	95%CI	*p*-value	HR	95%CI	*p*-value
SCS grade	3.012	1.001–9.059	0.049	2.626	1.224–5.637	0.013
Silva system	0.359	0.031–4.107	0.410	0.805	0.160–4.039	0.792
LNM	1.462	0.181–11.791	0.721	2.041	0.853–4.880	0.109
FIGO stage	1.883	1.067–3.325	0.029	1.072	0.364–3.158	0.900
DOI	1.919	0.510–7.219	0.335	1.382	0.509–3.753	0.525

*HR* hazard ratio, *CI* confidence interval

## Discussion

EAC is the second most common cervical cancer among women after cervical squamous cell carcinoma with increasing incidences. Recently, the Silva classification system of EAC has been proposed based on the pattern of invasion and has been widely acknowledged including National Comprehensive Cancer Network (NCCN) guidelines [32]. The value of the Silva system for predicting LNM and choosing surgical procedure in EAC patients has also been demonstrated, and was added to the 5th edition WHO classification of Female Genital tumours [30]. Considering the situation of intratumour heterogeneity, here we propose a scoring system aimed to explore a possible of a batter prognostic tool on the basis of the Silva system. Besides, there were studies focused on the schemes for further subgroup stratification, especially for Pattern C. This research also offers a fresh perspective on this topic.

### Guiding value of Silva system

In this study, we adapted the Silva system written in the latest 5th WHO classification and showed that the new pattern-based classification definitely plays an important role in prediction of LNM and selection of surgical procedures. Our results confirm the findings from previous studies that DOI, LVI, FIGO stage and LNM were significantly associated with the Silva system. The lymphadenectomy may be unnecessary for patients with pattern A because that no LNM were identified in pelvic lymph node. Considering the low rate of LNM in patients with pattern B, we believe that sentinel lymph node (SLN) mapping or a limited lymph node sampling should be sufficient in the absence of evidence of clinical LNM. Alternatively, or in addition, to up take the recommendations from the International Society of Gynecological Pathologists (ISGyP) that the procedure performed only in patients who are diagnosed as LVI positive in pattern B [11]. For patients with pattern C, considering the relatively higher risk of LNM, radical lymph node dissection should be indicated. In general, in this study we verified that the Silva system can be used to guide surgeons in choosing a method of operation.

However, although the survival curve of patients with pattern A appears to indicate a slightly better survival than the other two cohorts, no statistical difference was observed. The differences between OS and DFS estimated by Kaplan-Meier curves did not confirm prognostic value of the Silva system for OS or DFS in our study. Four studies provided information on OS and DFS in EAC patients using univariate analysis and multivariate analyses, and showed different conclusions. Byun et al. [15] analyzed 76 EAC patients and reported that Silva pattern remained a significant independent predictor of DFS. In this research, the 5- year survival rates for pattern A and pattern B were all 100%. Another study [18] reported that Silva system was a significant predictor of recurrence-free and disease-specific survival on a cohort of *n* = 82 usual type EAC. In contrast, Wang et al. [14] studied the clinicopathological data of 191 EAC patients from 3 medical centers, and indicated that Silva system is associated with recurrence-free survival, but found no correlation between Silva system and OS. Similar conclusions have been reported by Shi et al. [20], which suggest Silva system was only associated with tumour relapse. The current studies which evaluated the duration of survival had relatively small number of samples, and the results therefore considered to be ungeneralizable. Although some other studies numerically reported data of outcomes, they did not perform survival analysis [11, 33]. Alvarado-Cabrero et al. [11] reviewed the literature reporting on the subgrouping of EAC by Silva system and outcome, and the retrospective evidence supports the use of the Silva system for the clinical management of patients with EAC. Despite larger scale studies or longer follow-up periods are required to confirm the difference of survival time between different Silva pattens, the data for mortality can reflect the situation to some extent. Overall, the Silva system provide some prognostic guidelines but not always perfect.

The relationship between infiltration patterns and prognosis is complex [34]. Cancer invasion and metastasis is widely considered to be associated with stromal cells nearby the growing tumour [35]. Histopathology remains the gold standard of tumour diagnosis, infiltration patterns reflected the histomorphological appearance of microenvironment in tumours to some extent. Considering the heterogeneity of tumour, as well as the predictive value of LVI, such results of survival analysis in our study might be explained.

In order to predict the prognosis more precisely, a recent study by Shi et al. [20] developed a novel grading system based on tumour budding activity and cell cluster size in EAC. The results indicated that the grading system appears to be superior to the conventional FIGO grading and Silva pattern classification. However, tumour budding is merely one aspect of complex morphologic manifestations in cancer. In contrast, multiple criteria were collectively considered by Silva system and partly reflected the existence of tumour budding.

### The design of SCS and its potential prognostic value

The use of an effective risk and survival prediction system in any cancer is critical to patients and physicians for making decisions regarding adjuvant therapy modalities and the frequency of follow-up [36, 37]. In the existing criteria of Silva system, only the worst type is recorded when the tumours present with a mixed morphological pattern, rather than all signs together (i.e. tumours with pattern B and focal pattern C, should be classified as pattern C) [11]. The worse pattern is the one that is the most important factor to influence the prognosis, such a strategy in preoperative diagnosis is necessary and could avoid inadequate surgery.

However, this kind of evaluation modality can’t characterize the differences of complex appearances, which is becoming increasingly important in personalized treatments. The use of an effective risk and survival prediction system in any cancer is critical to patients and physicians for making decisions regarding adjuvant therapy modalities and the frequency of follow-up. As is explained in detail in the methods section, we propose a score system in this study, which defined as the sum of the two most common Silva morphologies and reported as SCS. Stolnicu S et al. [28] showed that LVI is also significantly associated with clinical outcomes, especially in tumours with early stage. Researches of LVI in EAC gave us a significant hint to note that LVI should be taken into consideration. SCS takes mixed morphologies and LVI status into account which has certain reference value for clinicians in evaluating the prognosis of OS and DFS with patients.

Accurate morphological evaluation has been demonstrated to be invaluable for helping with tumour treatment, especially in dealing with the heterogeneous components in neoplasms [38]. It is being discovered that the infiltration patterns provide precious hints concerning behavior of tumours, tumour heterogeneity was considered by a number of researches [34, 39]. It has recently been proposed that infiltration pattern predicts metastasis and progression better than the T-stage and grade in pancreatic neuroendocrine tumours, which also documents that more than 50% of the cases present intratumoural heterogeneity [39]. Similarly, different percentages of pattern in lung adenocarcinoma or prostate cancer should be reported.

It showed that 37.5% of the cases were mixed morphologies in this study, this suggests that the conception of intratumoural heterogeneity in EAC should be introduced to Silva system [22]. Here we need to point out that the description of “mixed” was only based on morphological phenomena to explore heterogeneity of the tumour, rather than as a substitute or a subversion for the concept of Silva system. The exploration of SCS does not affect the diagnosis of Silva system. Multivariate survival analysis of DFS and OS both suggested that SCS system was independently associated with survival in this study, while Silva system was not. The SCS system has better prognostic performance than the Silva system for predicting the prognosis of patients with EAC.

**Fig. 3:**
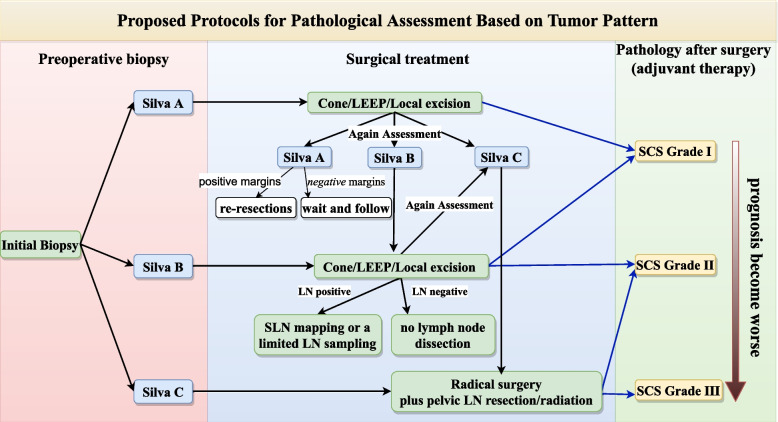
Proposed protocols for pathological assessment based on tumour pattern of EAC. Preoperative biopsy is shown at the left (red region), the surgical procedure is shown in the center (blue region), and postoperative pathology (green region) is shown at the right

Despite the promising results of our study, we must acknowledge that this study is only a preliminary exploration, and refinement of morphologic parameters will likely also improve the prognostic accuracy. In any case, this study provides initial evidence that morphological diversity could have clinical implications. In view of biopsy specimens would be a smaller size and the diagnosis always determined further surgical strategy, it may be more useful in specimens obtained during surgery which more accurately reflect the pathological features of the tumour than biopsy. As for biopsy specimens, we contend that the Silva system remains the best option (Fig. 3).

### A new idea for further typing pattern C

Previous studies [15, 40] have showed that pattern C accounts for 40.79–79.47% in the patients with EAC and our findings are largely consistent with the data reported in the literature. This proportion suggests that a large number of patients was diagnosed as pattern C and should underwent radical surgery and lymph node dissection. However, some tumours with pattern C are less aggressive, which could not effectively identified by the initial Silva system. It suggests that further stratification studies for pattern C may be required. There have been several different schemes to further distinguish tumours with pattern C in the existing literatures. A number of indices have been proposed such as the presence of LVI/LNM [13, 28], diverse morphologies (e.g., diffuse destructive, confluent, solid) [16, 24].

In our study, patients with pattern C were divided into two groups (SCS grade II, SCS grade III). It provides a new thought to further distinguish pattern C patients in different prognosis or different probability of LNM, that is, to group them in accordance with the presence of a milder morphology (pattern A or pattern B) area.

### Viewpoint at molecular level

Further advances on the basis of our study are still needed, especially deep viewpoint at molecular level. Analysis at genetic level can improve reliability of tumour classification, and it must deal with the concept of intratumoral heterogeneity, which is at the origin of tumour progression and is the byproduct of the selection process during the clonal expansion and progression of neoplasms [41]. Anjelica Hodgson et al. [42] present an important relationship between Silva system and specific oncogenic mutations for the first time. The study revealed a significantly higher prevalence of oncogene and tumour suppressor gene abnormalities in pattern B and C invasive EAC. This conclusion was validated by another research [18], which may suggest a tumour progression model in which mutations accumulate as the tumour invades in the surrounding stroma.

Researches of Silva system at molecular level showed a key underlying mystery that we should continue to explore. Here we assume that tumours exhibiting morphologic heterogeneity may be molecularly heterogeneous neoplasm, and we share aspiration that laser capture microdissection for next-generation sequencing analysis can be applied to explore the problems of SCS.

### Limitations

In this paper, we present a second-generation system based on the original Silva system called SCS, which was provided evidence for the potential value to predict OS and DFS in EAC patients. For the future goal of developing an improved risk prediction system by pathological factors, a larger sample size is needed. There were only eleven (9.2%) cases diagnosed as pattern A in our research, which is a lower rate range than that described in the literature (13.1–38.5%) [24, 43]. A relatively small proportion of pattern A probably resulted from the inclusion criterion in our study, with the inclusion of cases underwent radical hysterectomy only. Some patients with potential pattern A may have been excluded due to lymphadenectomy was not performed. Furthermore, the results and clinical implications of this study need to be validated in a prospective manner.

## Conclusions

In conclusion, the guided role of the Silva classification system for surgical options was further validated in our study. In this study, we focus on the prediction of OS and DFS, and provides evidence for the value of SCS which take the combined features and LVI into consideration to predict OS and DFS in EAC. Compared with original Silva system, SCS system we proposed provides an improved method for evaluating prognosis in EAC. The SCS system should prove useful for more precise of therapeutic trials in EAC and provided a new idea for further typing pattern C.

## Data Availability

The data that support the findings of this study are available from the corresponding author upon reasonable request.
